# Timing Is of the Essence: Improvement in Perception During Active Sensing

**DOI:** 10.3389/fnbeh.2019.00096

**Published:** 2019-05-09

**Authors:** Miguel Concha-Miranda, Javier Ríos, Joaquín Bou, Jose Luis Valdes, Pedro E. Maldonado

**Affiliations:** ^1^Laboratory of Neurosystems, Neuroscience Department, Faculty of Medicine, Universidad de Chile, Santiago, Chile; ^2^Faculty of Medicine, Biomedical Neuroscience Institute (BNI), Santiago, Chile

**Keywords:** active sensing, psychophysics, sensorimotor coordination, endogenous processes, active behavior

## Abstract

Active sensing refers to the concept of animals perceiving their environment while involving self-initiated motor acts. As a consequence of these motor acts, this activity produces direct and timely changes in the sensory surface. Is the brain able to take advantage of the precise time-locking that occurs during active sensing? Is the intrinsic predictability present during active sensing, impacting the sensory processes? We conjecture that if stimuli presentation is evoked by a self-initiated motor act, sensory discrimination and timing accuracy would improve. We studied this phenomenon when rats had to locate the position of a brief light stimulus, either when it was elicited by a warning light [passive condition (PC)] or when it was generated by a lever press [active condition (AC)]. We found that during the PC, rats had 66% of correct responses, vs. a significantly higher 77% of correct responses in AC. Furthermore, reaction times reduced from 1,181 ms during AC to 816 ms during PC For the latter condition, the probability of detecting the side of the light stimulus was negatively correlated with the time lag between the motor act and the evoked light and with a 38% reduction on performance per second of delay. These experiment shows that the mechanism that underlies sensory improvement during active behaviors have a constrained time dynamic, where the peak performances occur during the motor act, decreasing proportionally to the lag between the motor act and the stimulus presentation. This result is consistent with the evidence already found in humans, of a precise time dynamic of the improvement of sensory acuity after a motor act and reveals an equivalent process in rodents. Our results support the idea that perception and action are precisely coordinated in the brain.

## Introduction

During free behavior conditions, the changes in activities in the sensory organs are, more often than not, the result of self-initiated actions. This phenomenon has been termed “active sensing” (Bajcsy, 1988; König and Luksch, 1998) to contrast it with the traditional “passive sensing,” which is the perceptual process that results from the response to a sudden, unpredicted stimulus. For instance, active sensing occurs when there are changes in retinal activity, as a result of the saccadic movements of the eyes (Ito et al., 2011). Something similar occurs with the perception of touch, where this sensory modality requires the active movement of the hand (Gibson, 1962; Lederman et al., 1987), or as it occurs in the somatosensory system of rodents, where the movement of the whiskers is essential for an appropriate perception of the same (von Heimendahl et al., 2007; Prescott et al., 2011). This relationship between the motor act and sensory activity has already been reported in several studies (Melloni et al., 2009; Wurtz, 2015), and has also been performed using other modalities such as olfaction (Wachowiak, 2011), audition (Morillon et al., 2015) and somatosensation (Blakemore et al., 2000). These studies suggest that active sensing is a generalized mechanism of perception (Ulanovsky and Moss, 2008; Stamper et al., 2012; Hofmann et al., 2013; Schubert et al., 2015). However, the neurobiological mechanisms of the motor coordination between movements and perception, are still unknown. In a recent study on visual perception using free viewing and natural images, we found that the occipital evoked potentials to visual fixations and saccade onsets were larger, as compared to the same flashed stimuli (Devia et al., 2017). These results showed that stimuli for eye movements elicited stronger responses in visual areas than the equivalent “passive viewing” stimuli. Another study, conducted by Ito et al. (2011) showed that there are strong local field potential (LFP) modulations, coupled with the onset of saccades, that have greater amplitude than the equivalent LFP elicited by fixations. This study suggested that the LFP modulations observed in V1 during eye movement are more strongly coupled to saccade onset, than to the fixation period. Consistent with this interpretation, visually induced spikes, particularly the first spikes after the end of the eye movements, are locked onto a specific epoch of the LFP modulation observed after saccades. These studies suggest that the modulation of neural excitability, elicited from eye movements, may serve as a signal enabling the precise timing of spikes in the visual cortex and thereby provide a mechanism for spike synchronization. More generally, these results demonstrate that during active vision, the nervous system engages a mechanism of sensory modulation that is precisely timed to the self-initiated stimulus changes (Devia et al., 2017).

In addition, this principle where motor actions modulate sensory neural activity in a coordinated manner, has been shown to occur in rodents as well, in different sensory modalities (Petreanu et al., 2012; Vinck et al., 2015; Nelson and Mooney, 2016; Pakan et al., 2016, 2018; Dadarlat and Stryker, 2017; Leinweber et al., 2017; Itokazu et al., 2018). During whisker movements, there are motor related signals on S1 carried through M1; direct axonal projections that modulate the incoming sensory stimulation produced by the same whisker movement (Petreanu et al., 2012). Similarly, during rat eye movements, there are M2s to visual areas projections that are active during saccades (Itokazu et al., 2018). This same cortical area (M2), also conveys visual flow-related activity to V1 during locomotion (Leinweber et al., 2017). This is done through direct axonal projections from neurons that are especially sensitive to the sensory flow induced by movement, changing their activity depending on whether they are forming a closed loop with the sensory stimulation. Also, M2 projects to A1 in mice (Schneider et al., 2014; Nelson and Mooney, 2016), and its activation is associated with an inhibition of the auditory cortex response to stimuli. This has been interpreted as a mechanism for the attenuation of non-relevant auditory signals associated with movement, which has been recently observed at a behavioral level (Schneider et al., 2018). This evidence suggests that during sensorimotor interactions with the environment, there are neural modulations associated with the movement over sensory cortices, which are precisely coordinated in such a manner that the incoming stimuli activate the sensory areas, concurrently with these self-generated motor signals. Nevertheless, so far is still unknown to what extent this motor to sensory modulation mechanism requires precise coordination between the motor act and its sensory consequences. Is then the brain able to take advantage of the precise time-locking between the motor acts and the ensuing sensory activity that occur in active sensing? We hypothesize that during natural behavior, this precise coordination, between the self-generated actions and the consequential sensory activity, results in a more accurate sensory operation. Here, we report a behavioral study on rats where they had to locate the position of a brief light stimulus, either when it was elicited passively or generated by an active, self-initiated motor act. We found that the rats performed significantly better in the active than in the passive condition (PC), along with a reduction in reaction times, and that this improvement was dependent on the precise coordination between the motor act and the incoming stimulus. Our results support the idea that precisely coordinated action and sensory input impact perception competence.

## Materials and Methods

### Animals and Housing Conditions

All animal procedures were performed in accordance with the National Institutes of Health guidelines and were approved by the Universidad de Chile Faculty of Medicine Bioethics Committee on Animal Research. Fifteen adults, male Sprague-Dawley rats, with an average weight of 270–376 g, were housed in individual cages with inverted light-dark cycles and free access to food. Their water availability was restricted to 1 h per day. During the weekends the rats had free water and food access.

### Behavioral Training

**Figure 1:**
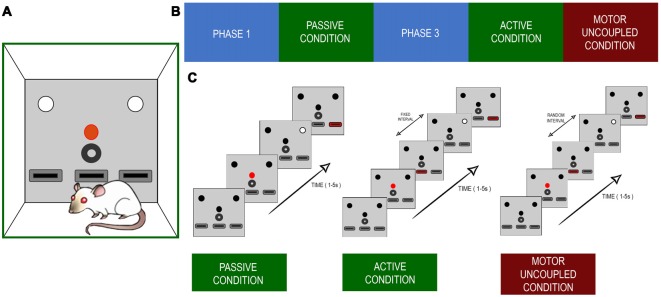
**(A)** Experimental setup. The configuration of lights and levers placed in the front panel of the Skinner box. The red circle depicts the red light that initiates a trial. The white circles indicate the lights cuing which of the left or right levers (black rectangles) is the one to be pressed for a reward. The black circle indicates the reward tube. The lower black boxes represent the levers. The central lever was the one the rats pressed during active and uncoupled conditions. **(B)** The sequence of training and recording scheme. **(C)** Time sequence for each of the experimental condition. The main difference between active and decoupled conditions was that the white light indicating the target lever appeared with a random delay, with respect to the onset of the central lever press.

The whole training program consisted of five phases. In phase one of training (PHASE1), the rats learned the stimulus light contingencies, in phase two the rats learned the PC, in phase three (PHASE3) they learned to turn on the stimulus light themselves, in phase four the active condition (AC) was presented (AC), and in phase five there was the motor uncoupled condition (MUC; Figure 1A). In PH1, each animal was subjected to a 30-min training session every weekday in operant conditioning boxes that had a set of three LEDs, each over one of the three levers, with a water dispenser in the central position (Figure 1B). Each trial began with a 500 ms activation of the central LED (warning light). Following that, 500 ms later, a second light, the stimulus light, was turned on either at the right or left side of the box. The light stayed on until the rats pressed any of the lateral levers. They were rewarded if they pressed the lever that was just under the stimulus light. The reward consisted of one drop of water sweetened with commercially available *Stevia rebaudiana* (0.02% w/v; Figure 1C). The trials were repeated until the daily training session was completed. Behavioral performance was estimated using the success rate (percentage of correct responses). When the rats achieved success rates of >70%, they moved onto the next training phase. In this second phase, the PC, the stimulus light was turned on, which lasted for 150–1,500 ms, randomly, among 10 possible values (150–1,500 ms in 150 ms steps), to present different levels of difficulty to the animal without changing any visual property of the stimulus. To be rewarded in this condition, the rats had to press the lever exclusively within a response window of 5 s after the stimuli light was turned off; otherwise, the reward was not delivered, and the next trial would begin. This occurred when the rats either pressed the opposite lever or pressed it before the stimulus light was turned off, or when they did not press any lever during the response window. In this condition, the rats were trained daily for 160 trials, to present the same number of stimuli for several possible durations. When the rats had achieved success rates of >70% and enough data had been gathered to estimate psychophysics curves, the rats were moved on to the next training phase. In PH3, the warning light started the trial as before, but the stimulus light was turned on only after the animal pressed the central lever after the warning light went off. Subsequently, the light stayed on, just as in PH1, and the rats had to press one of the lateral levels to continue to the next trial. When they had achieved success rates of >70% they were passed onto the next phase. In this fourth phase, AC, the rats had to press the middle lever (same as PH3) to turn on the stimulus light, but, in this case, for a variable duration (150–1,500 ms), as during PC. The rats then had to answer during a 5 s response windows. Finally, in the MUC stage, they had to repeat the same behavioral sequence as during AC, but the time interval between the first lever press and the stimulus appearing was at random, between 0 and 500 ms (uniformly distributed), and the duration of the stimulus was kept constant at 100 ms. This duration was chosen to avoid any ceiling effect during the task, as rats performed well when self-eliciting the stimulus even for the 150 ms light. Also, the duration was kept constant because otherwise there were too many numbers of combination of stimulus duration and lever-stimulus intervals to perform any robust statistics. During every training phase, the failure to respond, incorrect responses, and lever pressings during stimulus presentation were punished with variable time-outs. This training schedule allowed us to compare three behavioral conditions, PC, AC, and MUC. As a control experiment, three rats performed PC sessions after finishing the AC phase. The following section depicts a resume of each condition, describing the different trial outcomes that were evaluated.

### Task and Trials Outcomes

#### Passive Condition (PC)

After the warning light was turned off, a random lateral light was switched on for a variable time (150–1,500 ms), not requiring any action from the subject. The reward was delivered if the lever under the corresponding lateral light was pressed. All 15 rats performed this task, and the psychophysics curves were constructed using this data.

#### Active Condition (AC)

After the warning light was turned off, the rat was required to press the central lever to immediately activate (delay: 0 ms) a random lateral light of variable duration (150–1,500 ms). The reward was delivered if the lever under the corresponding lateral light was pressed. Seven rats performed this task, and the psychophysics curves were constructed using this data.

#### Motor Uncoupling Condition (MUC)

After the warning light was turned off, the rat was required to press the central lever to activate, after a random delay (0–500 ms), a random lateral light of 100 ms duration. The reward was delivered if the lever under the corresponding lateral light was pressed. The same seven rats of the AC performed this task.

For all the tasks, a correct response was defined as a correct lever press in the appropriate time window, a wrong response as an incorrect lever press in the appropriate time window, an interruption as pressing any levers before the stimuli light was turned off, and an omission as no levers being pressed until the response window was timed out.

### Statistical Analysis

Responses and reaction times were analyzed during each of the three conditions: passive, active, and motor uncoupling. During the passive and active conditions, four types of responses were consigned: omissions (OM), when the animal did not press any lever within the response interval; interruptions (IR), when the animal pressed a lever after the warning light and before the response interval; correct responses (CR), when the rats properly pressed the lever under the light stimulus during the response interval, and wrong responses (WR), when the rats pressed the lever opposed to the light stimulus. During CR, WR, and IR, the reaction times were always consigned. We excluded from analyses rats that did not reach 60% level of performance (considering only correct and wrong responses).

Psychophysics curves were constructed by calculating the probabilities of correct responses for each possible stimulus duration. This was performed after collapsing the responses from all sessions for each rat, which resulted in a single pool of responses for each animal. We then performed a one-way analysis of variance (ANOVA) for the probability of correct responses, with stimulus duration as a factor (10 levels, 150–1,500 ms). This probability was estimated for each stimulus, as the proportion of correct responses, after a particular duration, among all the correct and incorrect responses associated with the duration. A further ANOVA analysis, *t*-test, *post hoc* test was performed and corrected for multiple comparisons using the Holm-Sidak method.

The reaction times were analyzed as a function of the stimulus duration. A linear regression model was adjusted with the mean reaction time of each animal as the dependent variable and the stimulus duration as the independent one. One model for correct and one for wrong responses were performed independently.

IRs and OMs were analyzed similarly to the correct responses. The proportion of IRs and OMs were estimated for each stimulus duration as the number of them a rat performed (during a trial with a specific stimulus duration), divided by the total number of responses (interruptions, omissions, correct and wrong). We then performed a one-way ANOVA for both OMs and IRs, with stimulus duration as a factor.

The next aim was to compare passive vs. active conditions with a subgroup of the trained rats. A two-way ANOVA for CR, WR, OM, IR, with stimulus duration and condition (active or passive) as factors, was performed. Reaction times were compared in an analogous manner. Furthermore, a logit model to control for possible confounding factors was utilized. In fact, as the sessions occurred on different days, it was possible that any difference found in performance could be explained by learning. This means that rats would perform better after several days of training, irrespective of the condition of the task (active or passive). To control for this confounding factor, we performed a logit model with the probability of correct responses as the dependent variable and three factors as the independent variables; condition (passive or active), days of training, and stimulus duration. If the condition is the relevant factor explaining the differences in performance and not learning, then adding the number of training sessions as a third factor should lead to similar conclusions as on the ANOVA analysis (i.e., condition and stimulus durations should remain significant factors). To perform this second analysis, we included the data obtained during single sessions (instead of pooling data from all sessions per rat), where the rat neither necessarily answered every combination of stimulus duration nor performed correctly for most of the trials. Sessions where the rat failed to answer for more than two possible stimulus durations or did not reach 60% of the correct responses, was excluded from the logit model. As a second control of any learning effect, the performance of rats during all session, under passive (PC), active (AC) and training conditions (PH1 and PH3), was analyzed. The progression of performance, irrespective of the task, was analyzed as well, even during training conditions, to reveal any learning process that could better explain the improvement of performance during active conditions. To this end, we performed a linear regression model with the number of sessions as the independent variable, and performance as the dependent one.

### Regression Model for the Time Lag Between Motor Act and Stimulus

According to the time lag between motor initiation and the stimulus, the MUC responses were binned. Twenty bins of 25 ms windows to range the whole 0–500 ms interval was used, and an estimate of the proportion of correct responses of a particular bin was performed, pooling all trials that had a time lag between the motor act and the stimulus, within the corresponding time range. For these analyses, all the rats were pooled together to accumulate enough elements per time bin, and to achieve a more precise temporal definition. The reaction times were also analyzed as well as the amount of time the rats kept the lever pressed before releasing it (and then, trigger the stimulus) for each of the bins, to reveal if any observed change of performance could be explained by changes in these other variables. A linear regression model for these parameters was fitted, and the Pearson correlation between time lag and performance level was estimated.

## Results

**Figure 2:**
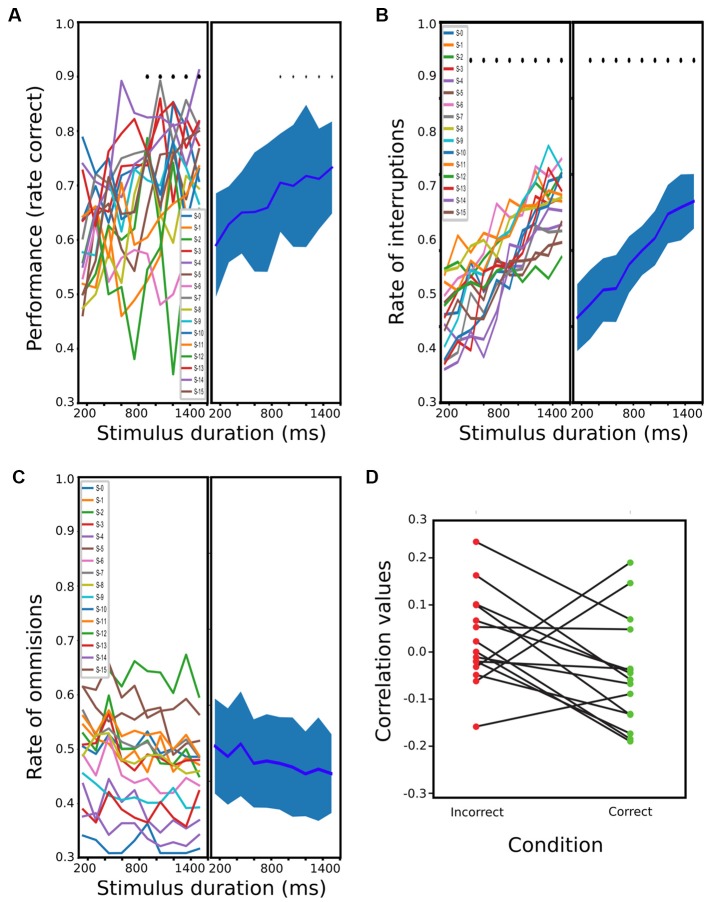
Rats performance during passive condition (PC). **(A)** Mean performance at different stimulus durations (from 150 ms to 1,500 ms). The bold line represents the mean, and the shading represents the standard error (s.t.d). The means and s.t.d are calculated using a single value of the rats’ mean performances at each stimulus duration, which results in 15 values (one per rat) for each possible stimulus. **(B,C)** same as **(A)** but for the proportion of interruptions and omissions, respectively. **(D)** Correlation values between reaction times and stimulus duration for each rat and for correct and incorrect trials (blue and red, respectively).

For PC, 15 rats were finalized after 20–30 sessions for the trained schedule. When plotting performance against stimulus durations, a dependence between the two variables can be clearly observed (Figure 2A). The one-way ANOVA revealed a significant effect of stimulus duration on performance (*df* = 9; *F* = 3.164; *p* < 0.0016), and a *t*-test further showed that the performance of stimuli lasting more than 900 ms was significantly better than during 1 of 150 ms (*t* = −3.87, −3.59, −4.02, −3.96, −5.16; *p* < < 0.0088, 0.0132, 0.0087, 0.0087, 0.0010 Holm-Sidak corrected, for stimuli between 900 and 1,501 ms). Regarding OMs and IRs, when plotting both variables against the stimulus duration, the number of OMs seems to decrease proportionally to the stimulus duration, while the interruptions showed a proportional increment (Figures 2B,C). A one-way ANOVA for these revealed a significant dependence between IRs and stimulus duration (*df* = 9; *F* = 28.5; *p* < 2.42 × 10^−28^) but not with OMs (*df* = 9, *F* = 0.73; *p* = 0.678). A *post hoc* *t*-test showed that the proportion of interruptions of all stimulus longer than 300 ms was significantly different from the 150 ms stimulus (*t* = −3.87, −3.59, −4.02, −3.96, −5.16 and *p* < 2.14 × 10^−03^, 8.72 × 10^−04^, 1.095 × 10^−03^, 9.59 × 10^−06^, 5.65 × 10^−07^, 5.32 × 10^−07^, 1.30 × 10^−07^, 1.21 × 10^−06^, 1.15 × 10^−07^, Holm-Sidak corrected, for stimuli between 300 and 1,501 ms). We then tested if the difference observed across stimuli duration on these behavioral parameters was accompanied by a change in reaction times. We calculated the Spearman correlation between reaction time and stimulus duration for each animal (Figure 2D), for both correct and wrong responses. We did not find any consistent relation between these two variables among rats. We also performed a 2-way ANOVA of the reaction times with correct and incorrect responses as first factor and stimulus duration as the second one. We found a significant effect of correct responses (*df* = 1, *F* = 7.67, *p* < 0.006) but not for stimulus duration (*df* = 9, *F* = 1.81 *p* = 0.0654), and no interaction (*df* = 9, *F* = 0.37, *p* = 0.948). *Post hoc* paired *t*-test showed that rats had shorter mean reaction times for incorrect responses (*df* = 298, *t* = −2.7614, *p* < 0.006).

### Active vs. Passive Conditions

**Figure 3:**
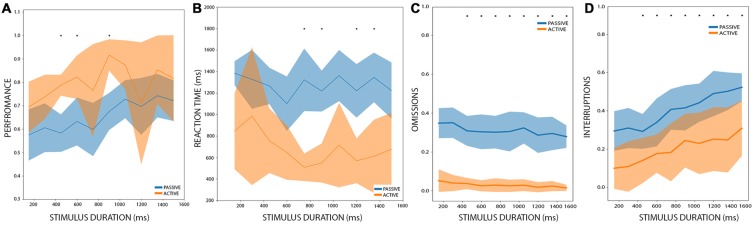
Performance comparison between PC and active condition (AC). **(A)** The performance of rats between AC and PC, during different stimulus durations. In orange: AC. Blue: PC. Bold lines represent the mean and the shaded areas correspond to the standard error. The mean and the s.t.d. are calculated for all rats. The asterisk represents significant differences between PC and AC after multiple *t*-test comparisons, with an alpha value of 0.05 (Holm-Sidak corrected). **(B–D)** are the same as **(A)** but for reaction times, omissions, and interruptions, respectively.

Among the 14 rats that performed the PC, seven rats reached the AC after another 15–20 sessions of training. We performed a 2-way ANOVA analysis with stimulus duration as one factor, and condition (passive or active) as the second factor. Stimulus duration and condition showed a significant effect on performance (stimulus duration: *df* = 9, *F* = 2.89, *p* < 0.004; condition: *df* = 1, *F* = 39.70, *p* < 5.1e-9) with no interaction (*df* = 119, *F* = 0.81, *p* = 0.60). When we pooled together all the stimulus durations and performed a *post hoc* *t*-test between the conditions, we found a significant improvement in active trials (*t* = 5.50, *p* < 0.0002). Now, when comparing each stimulus duration (Figure 3A), a multiple comparison *t-test* showed that in active conditions a rat performed better only during the 450, 600 and 900 ms stimulus durations (*t* = 6.85, 5.26 and 5.13; *p* < 0.048, *p* < 0.019 and *p* < 0.021 Holm-Sidak corrected, respectively). When looking at the individual performance of rats at each of these stimulus duration (Supplementary Figure S1), shows that their performance was consistent, and all the seven rats had improved their performance, whereas for the remaining duration, few rats exhibited an inverse relation (worsening their performance on active trials), probably explaining the higher and non-significant *p-values* in these cases.

We also performed a 2-way ANOVA analysis of the reaction times with stimulus duration and condition as factors. It showed that condition (passive or active) has a significant effect on reaction times (*df* = 1, *F* = 121.90, *p* < 6.0e-20) and that stimulus duration has no effect on reaction times (*df* = 9, *F* = 1.35, *p* = 0.21) with no interaction (*df* = 119, *F* = 0.68, *p* = 0.72), as can be observed in Figure 3B. The *post hoc* *t*-test demonstrated that during active conditions, rats have shorter reaction times (*t* = −3.02, *p* < 0.012). When comparing the reaction times for each condition at each stimulus duration, we found that in active conditions rats have shorter reaction times for the stimuli of 750, 900, 1,200 and 1,350 ms long (*t* = 6.10, 6.17, 5.67 and 5.73, *p* < 0.009, 0.009, 0.013, 0.013 Holm-Sidak corrected, respectively). This shows that in general rats perform better and have shorter reaction times under the AC.

Regarding interruptions and omissions, we performed a 2-way ANOVA analyses for these two variables, with stimulus duration and conditions as factors. Both factors affect the proportion of interruptions (stimulus duration: *df* = 9, *F* = 5.42, *p* < 2.28e-6; condition: *df* = 1, *F* = 92.87, *p* < 4.59e-17) with no interaction (*df* = 9, *F* = 0.24, *p* = 0.99). For omissions, only condition affects the proportion of omitted responses (stimulus duration: *df* = 9, *F* = 1.01, *p* = 0.43; condition: *df* = 1, *F* = 683.64, *p* < 9.89e-56) and there is no interaction between the factors (*df* = 9, *F* = 0.19, *p* = 0.99). A *post hoc* multiple comparison *t*-test (Figures 3C,D) resulted in a significant difference in the proportion of interruptions between the conditions for the 1,350 and 1,501 ms stimuli (*t* = 4.54 and *t* = 4.89, *p* < 0.024 and *p* < 0.018, respectively Holms-Sidak corrected). On the other hand, the proportion of omission shows a significant difference between all the stimuli (*t* = 8.14, 9.25, 9.96, 7.23, 7.49, 6.85, 8.93, 6.92, 6.63 and 10.5, *p* < 4.88e-4, 2.84e-4, 1.97e-4, 6.13e-4, 6.13e-4, 6.13e-4, 3.14e-4, 6.13e-4, 6.13e-4, 1.54e-4, Holm-Sidak corrected, for all 10 stimuli between 150 and 1,501 ms).

### Controlling for Learning as a Confounding Factor

**Figure 4:**
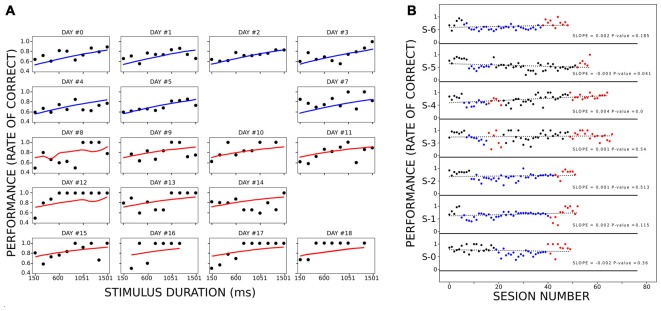
Performance of the rats when controlling for learning. Panel **(A)** represents the performance of the rats at each stimulus duration for different sessions. Within the panels, each black circle depicts the mean performance of all rats for a stimulus duration during an equivalent session. Each line (blue for PC and red for AC) corresponds to the fitted logit model for the corresponding combination of stimulus duration, session, and condition. The title of each panel shows the corresponding day of training (when day 9 is aligned with the beginning of the AC). **(B)** Performance of each rat during PHASE1 (black dots), PC (blue dots), PHASE 3 (black dots) and AC (red dots). Within each subpanel, the vertical position represents the proportion of correct responses within a session. The green dotted line is the linear model fitted for each series of dots. Each legend shows the slope of the linear model and the *p*-value of the *F*-test. Only S-4 had a linear model significantly different from a constant model.

To control for learning as a confounding factor that better explains the improvement of performance during active trials, we implemented a logit model of animal performance as a function of condition, stimulus duration, and number of sessions as factors (Figure 4A). We considered 20 sessions (occurring over 20 different days), eight from the PC and 12 from the AC, which included 1,974 trials in total. We considered session obtaining more than 60% correct responses, and less than four stimulus lacking responses (rats omitted or interrupted all trials with at least four different stimulus durations). Due to the latter reason, two training days were not included (days #6 and #15 of training). We fitted the model using the MLE method (log-likelihood = −1,110, *p*-value < 3.323e-11). Stimulus duration and condition (passive or active) had a coefficient significantly different from 0 (stimulus duration: *z* = 11.5, *p* < 1.25e-30; condition *z* = 2.80, *p* < 0.005), while the number of sessions did not (*z* = 1.70, *p* = 0.088). The odd ratio for stimulus duration (the exponential of the logit coefficients) was slightly above 1 (1.0010) compared to the condition odd ratio of 1.59, showing that among all the stimulus durations and conditions, the latter was more determinant on the animal’s performance.

Finally, we also analyzed the training sessions along passive and active conditions to reveal any learning processes during them, regarding the sensorimotor association between light and lever which could explain why rats perform better during AC. When plotting performance across the sessions (Figure 4B), only one rat showed a tendency to increase the proportion of correct responses across the session, irrespective of the condition (S-4. slope = 0.0039, *p* < 7.09e-7). When analyzing the improvement of the performance when removing this rat, the results stayed the same. The ANOVA analysis showed that performance depended on both stimulus duration (*df* = 9.0, *F* = 2.81 *p* < 4.98e-03) and condition (*df* = 1.0, *F* = 37.10, *p* < 1.41e-08). The *post hoc* analyses revealed a significant increase of performance during AC as compared to passive, for 450, 600 and 900 ms stimulus durations (*t* = 7.20, 5.21 and 5.13; *p* < 0.003, 0.020 and 0.022, respectively). On a last control experiment, three rats performed a second phase of PC following the AC (Supplementary Figure S2). Rats on this last phase did not reach the maximum levels of performance that the one attained during the AC, although they improved over the training baseline. This fact was also observed in Figure 4B, where on AC rats reached higher performances than during PC. This fact cannot be explained by a sustained improvement of performance across days of training because when rats stayed on passive conditions for more than 30 consecutive sessions (rats 1, 2 and 6) they never reached as higher performance as on active trials (with a single exception on rat 5 on only one session).

### Improvement of Performance Depends on Temporal Expectations

**Figure 5:**
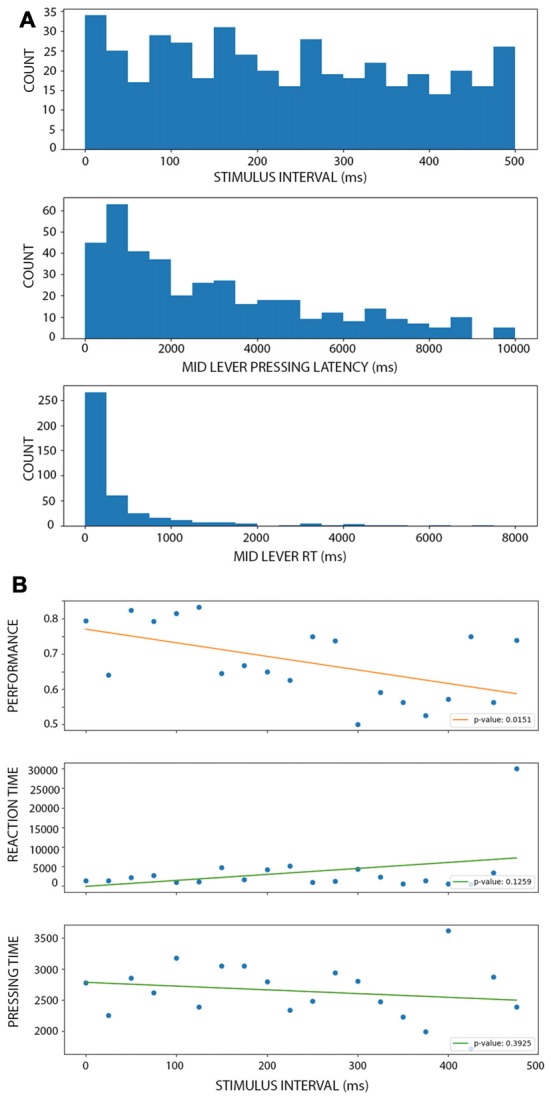
Performance of the rat during motor uncoupled condition (MUC). (**A**; from top to bottom) Histograms showing the number of trials with a stimulus interval (top), mid lever latency (middle) and mid lever reaction time (bottom) within the corresponding bin. There is a similar number of trials at each stimulus interval bin. (**B**; from top to bottom) Linear models of performance (top), reaction time (middle) and pressing time (bottom) as a function of the stimulus interval (when binning using 25 ms windows). Each dot represents the mean performance, reaction time or pressing time for trials within each bin. Each legend shows the *p*-value associated with the *F*-test, showing that only performance linearly depends on the stimulus interval.

To evaluate if the improvement of performance observed during active conditions depended on temporal expectations, we designed a third condition where the stimulus-evoked was temporarily uncoupled with the middle lever press. In these trials, a 100 ms stimulus appeared after the rats pressed the middle lever but after a random interval between 0 and 500 ms. This resulted in a uniform distribution of intervals (Figure 5A, upper panel). When binning the intervals at 20 ms duration, we obtained between 15 and 33 trials per bin. The middle panel depicts the distribution of the times the rats kept the lever pressed before releasing it (and then eliciting the stimulus). The lower panel shows the distribution of middle lever reaction times, that is, the time between the warning light and the middle lever press. As expected, longer intervals were associated with lower performance (Figure 5B, lower panel; linear model: *r* = −0.535, *p* < 0.016), with a 38% decrease in the performance per second of delay. In fact, after 300 ms of delay, the mean performance is 61.6% for the trials, which is as low as on the shortest stimulus in the PC (Figure 2A; which is also, the worst performance reached at the group level). When looking at the time duration the rats kept the lever pressed to the reaction times, neither of these variables showed a clear change associated with the time interval (Figure 5B, middle and lower panel). In fact, both linear models were not significantly different from a constant model (for lever press time: *r* = 0.35, *p* = 0.12; for reaction times: *r* = −0.20, *p* = 0.39), confirming that the change in performance cannot be directly explained by a linear change in these other variables. When comparing the performance during AC and MUAC there were no difference between conditions (*df* = 49, *t* = 0.0304, *p* = 0.97; Supplementary Figure S3).

## Discussion

The present work shows that it was possible to train rats to perform a sensorimotor task during passive and active conditions. They learned to discriminate the location of the light at different stimulus durations, having a greater number of correct responses and shorter reaction times for longer stimuli, while at the same time increasing the number of interruptions. Several behavioral parameters improved during the AC, as it was possible to observe a greater number of correct responses and shorter reaction times during the AC, as well as a decrease in the number of interruptions and omissions. The improvement of performance could not be explained by learning, as controlling for the number of training sessions through the days did not alter these results. Finally, the MUC experiment showed that the improvement of performance observed during the AC was dependent on the precise coordination between the motor act and the sensory stimulus.

Our results demonstrate that active behavior can improve behavioral performance in a sensorimotor task, with similar experiments showing that post a self-generated stimulus or during locomotion, there is an improvement of the rat’s sensory discrimination. In fact, on a similar paradigm but in a different sensory modality, Carcea et al. (2017) showed that after self-producing auditory stimuli, rats improved their auditory discrimination capabilities, as observed in decreased detection thresholds and through the tuning of their psychophysics curves, when compared to a PC. Similarly, although in a different experimental condition, Bennett et al. (2013) showed that during locomotion over a spherical treadmill, mice also improved their visual discrimination performance by increasing their probability to detect a drifting grating. Recently it has been also shown that movement has an attenuation effect over auditory stimulus, which is experience-dependent and acts as “filter,” impairing the detection of predictable stimulus while improving mice abilities to distinguish novel ones (Schneider et al., 2018). These few instances of improved performance are in marked contrast with the increasing evidence regarding the effect of movement and locomotion over sensory cortices at a single and population neural level (Busse, 2018). This implies, that despite it being shown that movement can improve cortical processing of sensory stimuli in several ways, it is still not clear how all these modulatory effects affect behavior, and which are the time constraints of them. The present work contributes in this direction, showing that the behavioral improvements observed during movement-evoked trials have a precise time dynamic, already decaying in a 500 ms time window, and that the enhancement occurs at several behavioral dimensions.

The mechanisms under these behavioral improvements may be diverse, as different neural movement-related modulations have been described in terms of the brain. One possibility is that movement induces a neuromodulator release of the sensory cortex (Nelson and Mooney, 2016) that has been shown to increase the gain of V1 neurons (Polack et al., 2013); consistent with the acute effect of topically administered acetylcholine in anesthetized rats (Soma et al., 2013). Another possibility is thalamocortical (Pakan et al., 2016), or cortico-cortical (Manita et al., 2015; Leinweber et al., 2017) modulation over sensory cortices. The later modulation has been observed on M2 axonal projections to sensory cortices, which participate in visual flow prediction (Leinweber et al., 2017), in accurate somatosensory discrimination (Manita et al., 2015), and in auditory detection as discussed above (Schneider et al., 2018). On this latter case, a close relation between M2 neural activation and behavioral improvements has been shown, mediated by an inhibitory local circuit in auditory cortex which can be plastically tuned during movement and activated by M2 neurons. A similar auditory cortex attenuating effect has been observed for lever press movements as the ones implemented on the present study (Rummell et al., 2016). These processes are not necessarily exclusive since both process, forebrain neuromodulation and M2 cortico-cortical direct connections, simultaneously influence sensory cortices during movement (Nelson and Mooney, 2016). A third alternative is that movement induces a higher level of arousal, which has also been shown to contribute to sensory processing, independent of locomotion (Vinck et al., 2015).

Additionally, there may be several endogenously generated brain processes, simultaneously occurring during active behavior, parallel to any direct motor effect on sensory cortices. It has been described that sensory processing is modulated by several top-down influences, such as expectation (Fiser et al., 2016) and attention (Kim et al., 2016; Zhang et al., 2016) and that top-down influences can regulate action selection (White et al., 2018). All these processes can, in principle, improve animal performance during the task, either by enhancing sensory processing or by facilitating action selection. Nevertheless, the present work shows that this enhancement is precisely timed and that then any postulated brain mechanism, as the ones discussed above, must follow a similar dynamic. We propose that the study of the precise dynamics under these processes will help disentangle the different brain mechanisms occurring during active behavior, as they range from short motor signals lasting just a few milliseconds, as during the corollary discharge (Schneider et al., 2014), to the neuromodulatory effect of arousal and locomotion that can last for seconds (Vinck et al., 2015; Nelson and Mooney, 2016).

## Ethics Statement

All animal procedures were performed in accordance with the National Institutes of Health guidelines and were approved by the Universidad de Chile Faculty of Medicine Bioethics Committee on Animal Research.

## Author Contributions

The experimental design was developed and the manuscript was written by MC-M, JR, JV and PM. The data collection was conducted by MC-M, JR and JB. The analysis was conducted by MC-M and JR.

## Conflict of Interest Statement

The authors declare that the research was conducted in the absence of any commercial or financial relationships that could be construed as a potential conflict of interest.

## Supplementary Material

FIGURE S1 Comparison of rat performances between passive condition (PC) and active condition (AC). Panel **(A)** shows the difference in mean performance between PC and AC when considering all trials and sessions of each rat for the two conditions. There is a significant difference (alpha = 0.05) between the two conditions. Panel **(B)** same as **(A)** but for reaction times, showing that there is a decrease in reaction times during AC. **(C)** Performance of rats between PC and AC at different stimulus duration. Each panel depicts the mean performance of each rat between the two conditions during the trials of stimulus duration, as expressed in the title of each panel. Panels with an asterisk indicate significant differences (as expressed on the main text). Figure 2A is built using these values. Panel **(D)** same as **(C)** but for reaction times.

FIGURE S2 Comparison between PC and AC, when PC follows AC. Each panel represents one different rat, where a boxplot of performance during AC and PC is depicted. The data was computed across multiple sessions.

FIGURE S3 Comparison between AC and MUC. Boxplot of mean performance during AC and MUC at each session (all rats pooled together). No significant differences were found (*df* = 49, *t* = 0.0304, *p* = 0.97).
