# Supplementary figures and images for: Timing Is of the Essence: Improvement in Perception During Active Sensing
Source: Front Behav Neurosci. 2019 May 9;13:96. doi: 10.3389/fnbeh.2019.00096 (PMC6520616; doi:10.3389/fnbeh.2019.00096)

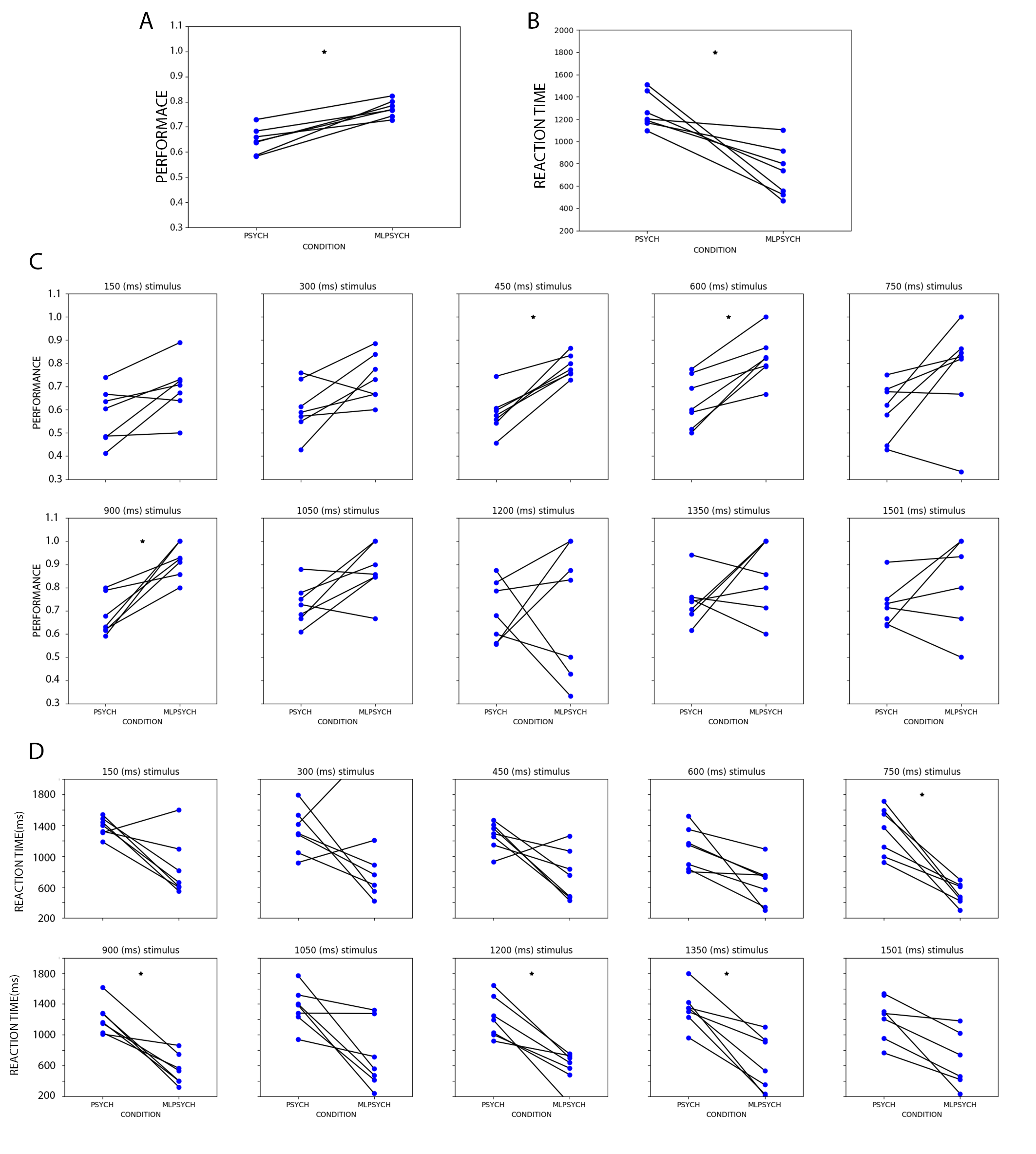

Supplement: FIGURE S1 — Comparison of rat performances between passive condition (PC) and active condition (AC). Panel (A) shows the difference in mean performance between PC and AC when considering all trials and sessions of each rat for the two conditions. There is a significant difference (alpha = 0.05) between the two conditions. Panel (B) same as (A) but for reaction times, showing that there is a decrease in reaction times during AC. (C) Performance of rats between PC and AC at different stimulus duration. Each panel depicts the mean performance of each rat between the two conditions during the trials of stimulus duration, as expressed in the title of each panel. Panels with an asterisk indicate significant differences (as expressed on the main text). Figure 2A is built using these values. Panel (D) same as (C) but for reaction times. [file Image_1.TIF]

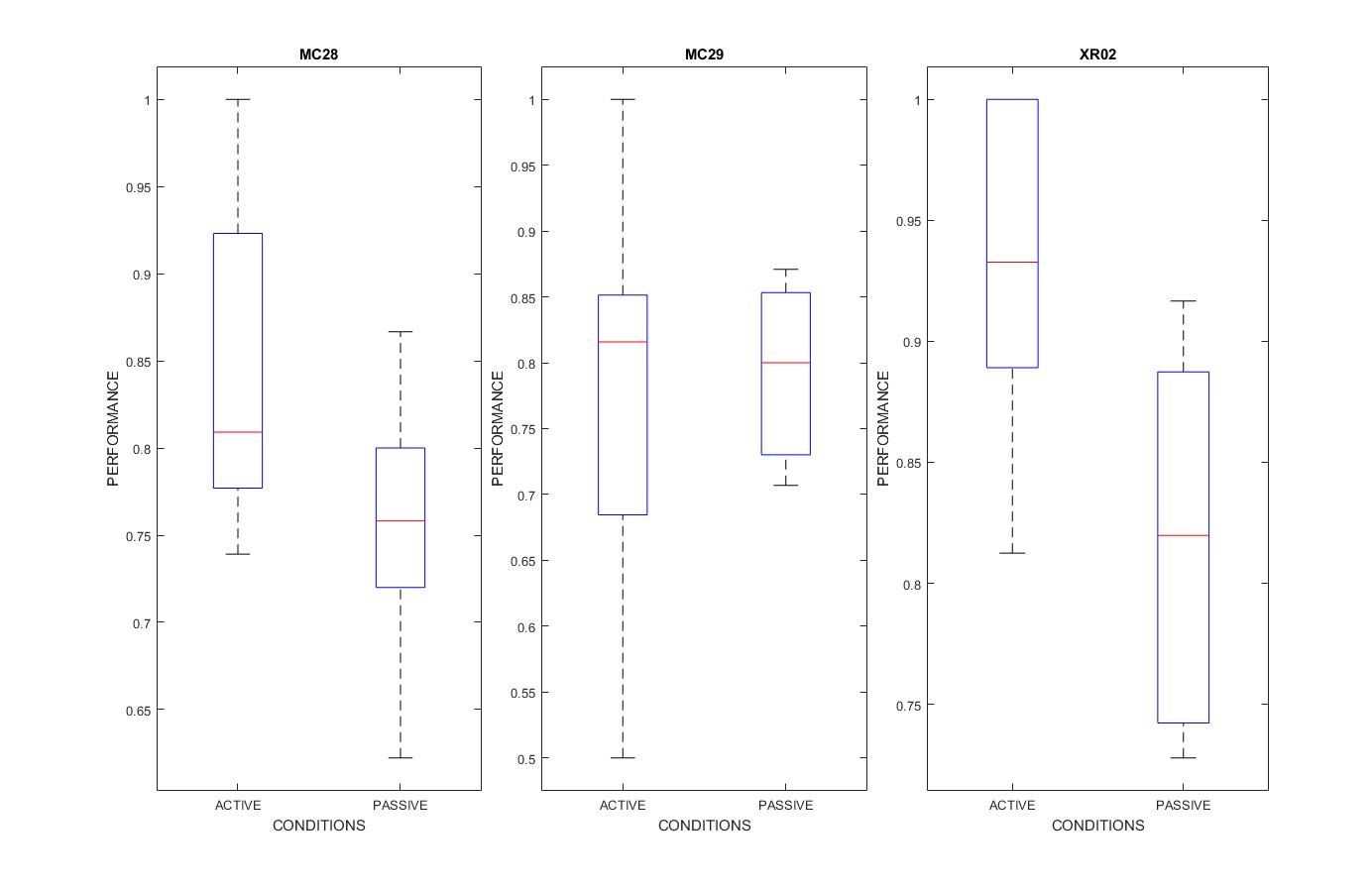

Supplement: FIGURE S2 — Comparison between PC and AC, when PC follows AC. Each panel represents one different rat, where a boxplot of performance during AC and PC is depicted. The data was computed across multiple sessions. [file Image_2.TIF]

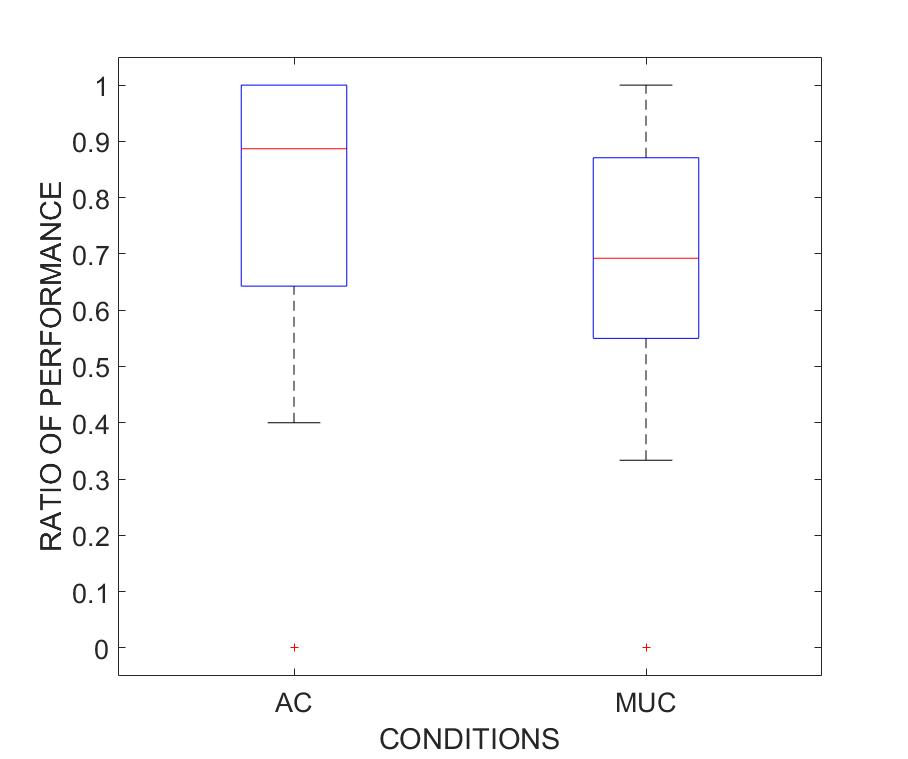

Supplement: FIGURE S3 — Comparison between AC and MUC. Boxplot of mean performance during AC and MUC at each session (all rats pooled together). No significant differences were found (df = 49, t = 0.0304, p = 0.97). [file Image_3.TIF]
